# Simulation-driven Selection of Electrode Materials Based on Mechanical Performance for Lithium-Ion Battery

**DOI:** 10.3390/ma12050831

**Published:** 2019-03-12

**Authors:** Abhishek Sarkar, Pranav Shrotriya, Abhijit Chandra

**Affiliations:** Department of Mechanical Engineering, Iowa State University, Ames, IA 50011, USA; asarkar@iastate.edu (A.S.); achandra@iastate.edu (A.C.)

**Keywords:** lithium-ion battery, mechanical stability, material index, parametric analysis, elasto-plastic stress

## Abstract

Experimental and numerical studies have shown that mechanical loading associated with lithiation/delithiation may limit the useful life of battery electrode materials. The paper presents an approach to parameterize and compare electrode material performance based on mechanical stability. A mathematical model was developed to determine particle deformation and stress fields based upon an elastic-perfectly plastic constitutive response. Mechanical deformation was computed by combining the stress equilibrium equations with the electrochemical diffusion of lithium ions into the electrode particle. The result provided a time developing stress field which shifts from purely elastic to partially plastic deformation as the lithium-ion diffuses into the particle. The model was used to derive five merit indices that parameterize mechanical stability of electrode materials. The merit indices were used to analyze the mechanical stability for the six candidate electrode materials—three for anode materials and three for the cathode material. Finally, the paper suggests ways to improve the mechanical performance of electrode materials and identifies mechanical properties that need to be considered for selection and optimal design of electrode materials.

## 1. Introduction

Increasing energy demand for high energy density storage devices makes lithium-ion batteries a prime source for energy storage [1]. Graphite/lithium cobalt oxide was the first electrode material [2] but rigorous experimental and theoretical study on high capacity and stable electrode materials have allowed lithium batteries to achieve higher energy density, longer cycle life, and safer operation [3]. Electrode performance has been studied through experiments, molecular dynamics simulation and multiphysics modeling [4]. Recent development has focused on finite element modeling for understanding the thermo-mechanical functioning of electrodes and search for newer battery materials of better mechanical and thermal performance at high charging rates [5,6].

The structure and mechanisms governing lithium-ion diffusion and storage vary for different electrode materials. Lithium manganese oxide (LMO) has a cubic spinel structure with the manganese located in the octahedral sites and the lithium ions occupying the tetrahedral sites in a cubic close-packed array of oxygen [7]. LMO gets oxidized in presence of lithium-ion and diffusion process is governed by the chemical potential across the electrodes [8,9]. The insertion of the lithium ions in the vacant octahedral locations produces the Jahn Teller distorted tetragonal phase. The lithium cobalt oxide (LCO) electrode has a α-NaFeO_2_ layered structure with a cubic close packed array of oxygen with cobalt and lithium ions occupying octahedral sites in alternating layers. Higher voltage applications could cause structural instability within the delithiated layers of LCO [10,11]. Olivine lithium ferrous phosphate (LFP) electrode has an orthorhombic structure with FeO_6_ octahedra and PO4 tetrahedra networked in a one-dimensional channel. These channels in LFP provide pathway for lithium to diffuse effectively. Graphite has a layered graphene sheets in either hexagonal (common) or rhombohedral stacking structure with lithium ions diffusing and intercalating between the graphene layers [12]. With development in nanotechnology, silicon nanoparticle-based electrodes have been developed as a promising anode material because of large energy storage capacity (3579 mAh/g) [13]. Silicon in the first cycle reacts with lithium to transform from crystalline to amorphous structure [14] and the amorphous silicon intercalates from then onwards. In the current analysis, amorphous lithiated silicon electrode has been considered. Lithium titanate (LTO) has a spinel structure with no strain during deformation during two-phase lithiation/delithiation process.

Mechanical stability of electrodes is crucial for life prediction of lithium-ion battery. Analytical models predicting the stability of lithium-ion battery electrodes have either assumed a perfectly plastic material or an elastic material [15,16]. Christensen et al modeled lithiation induced stress assuming that stress is a function of the lithium-ion concentration gradient in the particle [17]. Zhang et al [18,19], modeled the radial and hoop stress in ellipsoidal particles considering elastic deformation of lithium manganese oxide spinel. In contrast, perfect plasticity-based models have been developed for anode materials for silicon [15,20]. These models considered pure plasticity due to small impact of the elastic deformation. TEM analysis by Liu et al [21] has shown plastic deformation of 320% by silicon during lithiation. Current research is being focused on novel materials like layered-lithium nickel manganese cobalt electrodes (NMC), which have high capacity (>250 mAh/g) at high discharge potential (3.6–4.5 V) [22]. However, these electrodes are mechanically unstable in high potential domains. 

A rigorous mathematical model was developed for evaluation of elasto-plastic stress state in electrode materials. The model was used to study the stress evolution and fracture response in electrode particles of different materials. A set of five merit indices were created which parameterize the materials based on their mechanical performance and fracture stability. A detailed analysis of these indices provided an insight of the material properties useful for a performance boost of the electrode materials. Six electrode materials, three for the cathode and three for the anode, were selected for this study. The results discussed the electrode materials that stand out among the others for having the better mechanical stability and fracture resistance. The paper discusses the crucial material properties which influence the life of a battery, a set of merit indices to evaluate new materials and provides an approach to improve the mechanical performance of lithium-ion battery electrodes.

## 2. Mathematical and Parametric Analysis

### 2.1. Mathematical Model 

**Figure 1:**
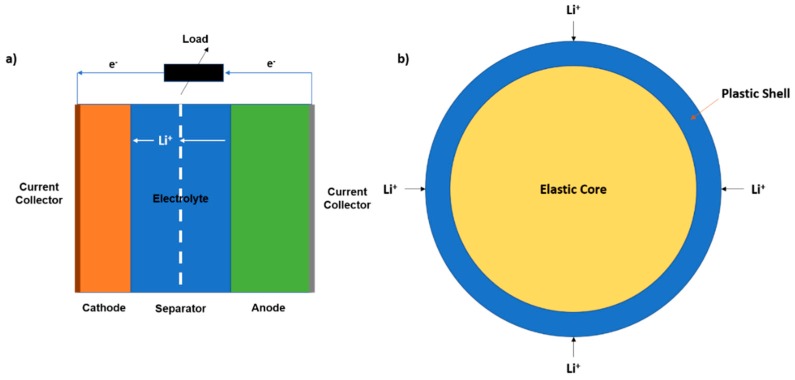
(**a**) Lithium battery schematic; (**b**) lithium cathode stress domains; during discharge cycle.

A lithium-ion battery works by the principle of electrochemical diffusion of lithium-ion due to a potential difference between the electrodes. As the cell discharges, the lithium ions diffuse into the cathode from the anode, thereby converting chemical energy into electrical work. Figure 1 and Equations (1) and (2) describe the reaction in any generic lithium transition metal during the discharging process.

(1) $$Li_{1-x}MO_{y}+xLi^{+}+xe^{-}\overset{cathode}{\to }LiMO_{y}$$

(2) $$Li_{x}A\overset{anode}{\to }xLi^{+}+xe^{-}+A$$

During discharge, lithium ions dissociate from the anode and intercalate with the cathode, made up of a metal oxide of lithium, to form a lithium intercalation compound and vice-versa during charging. Many mathematical models on the elastic deformation of electrode particle predicted the mechanical performance of certain electrode materials [18,19,23]. In the present work, the deformation of the electrode particle in the plastic regime was modeled. The porous electrode theory [17] with the electrolyte having an infinite source of lithium ion, free expansion of the particle surface and the perfectly plasticity of the material at yield point were assumed for modeling. The mass transport of lithium ions in the electrode material is expressed below where M is the mobility and c is the concentration of lithium-ion.

(3) J=−cM∇μ

The electrochemical potential (μ) is expressed as a function of lithium-ion mole fraction (x) and the chemical stress generated.

(4) $$\mu =\mu^{0}+RTln\left(x\right)-\Omega \sigma_{h}$$

$\sigma_{h}$ is the hydrostatic stress on the particle due to differential expansion, Ω is the partial molar volume, and x is the mole fraction of lithium in the particle. Substituting the electrochemical potential (Equation (4)) into mass flux (Equation (3)), the equivalent stress dependent ion flux equation was derived.

(5) $$J=-Mc\left[RT\frac{\nabla c}{c}-\Omega \nabla \sigma_{h}\right]=-D\left[\nabla c-\frac{\Omega c}{RT}\nabla \sigma_{h}\right]$$

D is the mass diffusion coefficient of the lithium-ion in the electrode, and T is the operating temperature. Fick’s Law of diffusion was expressed for a spherically symmetric particle from the diffusion mass flux (Equation (5)).

(6) $$\frac{\partial c}{\partial t}=D\left[\frac{1}{r^{2}}\frac{\partial }{\partial r}\left(r^{2}\frac{\partial c}{\partial r}\right)-\frac{\Omega }{RT}\left\{\frac{\partial c}{\partial r}\frac{\partial \sigma_{h}}{\partial r}+\frac{c}{r^{2}}\frac{\partial }{\partial r}\left(r^{2}\frac{\partial \sigma_{h}}{\partial r}\right)\right\}\right]$$

The following boundary conditions were used to solve Equation (6).

(7) $$i=\frac{\alpha \rho r_{0}}{3}C_{rate}$$

(8) $$J{|}_{r=r_{0}}=\frac{i}{F}$$

(9) $${\frac{\partial c}{\partial r}|}_{r=0}=0$$

Equation (7) shows the current flux (i) depends on the theoretical capacity of the electrode material (α), density (ρ), radius ($r_{0}$) and the rate of charging ($C_{rate}$). 

Equation (6) shows hydrostatic stress coupled with lithium-ion concentration, which affects the diffusion process. The intercalation of lithium-ion into the electrode causes an expansion of the particle. Particle expansion leads to shifting of atomic planes causing dislocation of atoms from their natural lattice sites. This leads to the generation of a stress field in the particle. Therefore, the formulation of the elastic component of strain was found similar to the strain produced during to thermal expansion [18].

(10) $$\varepsilon^{e}{}_{r}=\frac{1}{E}\left[\sigma_{r}-2\nu \sigma_{\theta }\right]+\frac{\tilde{c}\Omega }{3}=\frac{du}{dr}$$

(11) $$\varepsilon^{e}{}_{\theta }=\frac{1}{E}\left[\left(1-\nu \right)\sigma_{r}-\nu \sigma_{\theta }\right]+\frac{\tilde{c}\Omega }{3}=\frac{u}{r}$$

(12) $$\tilde{c}=c-c_{0}$$

The radial ($\varepsilon_{r}$) and hoop ($\varepsilon_{\theta }$) strain depended upon the concentration variation across the particle. u is the radial displacement and $c_{0}$ is the initial lithium-ion concentration. As the electrode was further lithiated, the equivalent stress exceeded the yield stress causing the material to deform plastically. For a spherical particle, the yield criterion follows the condition below.

(13) $$\left|\sigma_{r}-\sigma_{\theta }\right|\leq S_{y}$$

When the equivalent stress exceeds the yield limit ($S_{y}$), the total strain generated was due to both the elastic and plastic deformation of the particle. Figure 1b represents the elasto-plastic schematic of a cathode particle during lithiation. The total (hydrostatic) strain would be equal to the summation of the volumetric elastic and plastic strain.

(14) $$\varepsilon_{ij}=\varepsilon^{e}{}_{ij}+\varepsilon^{p}{}_{ij}$$

Since the particle was assumed completely spherical, the stress equilibrium equation was solved in the spherical coordinates considering radial symmetry.

(15) $$\frac{\partial \sigma_{r}}{\partial r}+\frac{2}{r}\left(\sigma_{r}-\sigma_{\theta }\right)=0$$

$\sigma_{r}$ is the radial stress component and $\sigma_{\theta }$ is the hoop stress component. This equation was solved for the elastic segment by substituting the elastic displacements and for the plastic part by substituting the yield equality using the following boundary conditions [24].

(16) $${\frac{\partial \sigma_{r}^{el}}{\partial r}|}_{r=0}=0$$

(17) $$\sigma_{r}^{el}=\sigma_{r}^{pl}{|}_{r_{p}}$$

These conditions satisfied the radial stress continuum at the core and interface between elastic and plastic domain ($r_{p}$). The plastic stress was solved by substituting the yield equality into the stress equilibrium equation with free expansion along the radial direction on the surface.

(18) $$\sigma_{r}^{pl}{|}_{r_{o}}=0$$

The stress (radial and hoop) was calculated by solving the elastic-perfectly plastic equations for the plastic domain.

(19) $$\sigma_{r}^{pl}=2S_{y}ln\left[\frac{r_{o}}{r}\right];r_{p}\leq r\leq r_{o}$$

(20) $$\sigma_{\theta }^{pl}=2S_{y}ln\left[\frac{r_{o}}{r}\right]-Y;r_{p}\leq r\leq r_{o}$$

The differential equation for the elastic domain was solved by using the plastic radial stress at the plastic interface.

(21) $$\sigma_{r}^{el}=2S_{y}ln\left[\frac{r_{o}}{r_{p}}\right]+\frac{2\Omega E}{3\left(1-\nu \right)}\left[\frac{1}{r_{p}{}^{3}}\int_{0}^{r_{p}}\tilde{c}r^{2}dr-\frac{1}{r^{3}}\int_{0}^{r}\tilde{c}r^{2}dr\right];0\leq r\leq r_{p}$$

(22) $$\sigma_{\theta }^{el}=2S_{y}ln\left[\frac{r_{o}}{r_{p}}\right]+\frac{\Omega E}{3\left(1-\nu \right)}\left[\frac{2}{r_{p}{}^{3}}\int_{0}^{r_{p}}\tilde{c}r^{2}dr+\frac{1}{r^{3}}\int_{0}^{r}\tilde{c}r^{2}dr-\tilde{c}\right];0\leq r\leq r_{p}$$

The mean (hydrostatic) stress was then found for the elastic and plastic equations.

(23) $$\sigma_{h}=\frac{\sigma_{r}+2\sigma_{\theta }}{3}$$

(24) $$\sigma_{h}^{pl}=2S_{y}ln\left[\frac{r_{o}}{r}\right]-\frac{2}{3}Y;r_{p}\leq r\leq r_{o}$$

(25) $$\sigma_{h}^{el}=2S_{y}ln\left[\frac{r_{o}}{r_{p}}\right]+\frac{2\Omega E}{9\left(1-\nu \right)}\left[\frac{3}{r_{p}{}^{3}}\int_{0}^{r_{p}}\tilde{c}r^{2}dr-\tilde{c}\right];0\leq r\leq r_{p}$$

The elastic and plastic stresses in Equations (24) and (25) were substituted into Equation (6) to decouple the concentration from stress.

(26) $$\frac{\partial c}{\partial t}=D\left[\frac{1}{r^{2}}\frac{\partial }{\partial r}\left(r^{2}\frac{\partial c}{\partial r}\right)+\theta {\left(\frac{\partial c}{\partial r}\right)}^{2}+\theta c\left\{\frac{1}{r^{2}}\frac{\partial }{\partial r}\left(r^{2}\frac{\partial c}{\partial r}\right)\right\}\right];0\leq r\leq r_{p}$$

(27) $$\frac{\partial c}{\partial t}=D\left[\frac{1}{r^{2}}\frac{\partial }{\partial r}\left(r^{2}\frac{\partial c}{\partial r}\right)-\frac{\Pi }{r}\left(\frac{c}{r}+\frac{\partial c}{\partial r}\right)\right];r_{p}\leq r\leq r_{o}$$

$\theta =\frac{2\Omega^{2}E}{9RT(1-\nu )}$ and $\Pi =\frac{2S_{y}\Omega }{RT}$ are constants which coupled the gap between stress driven concentration and diffusion driven concentration in the elastic and plastic equations, respectively.

Although fatigue failure of lithium-ion electrode is a more dominant mechanism than fracture, but the ability of the material to prevent crack propagation is a primary characteristics needed to avoid the onset of failure. A simple fracture analysis was used to compare different electrode materials based on their material toughness and ability to prevent crack propagation upon loading. The fracture of the spherical electrode during lithiation occurs due to the hoop stress. The simplest model for an edge crack relates the stress intensity with the applied stress and crack size.

(28) $$K_{I}=C\sigma_{\theta }\sqrt{\pi a}$$

a is the crack depth from the surface, $K_{I}$ is the stress intensity factor and C is a geometric factor.

### 2.2. Material Characterization for Lithium Electrodes

The life of the battery depends on several parameters of which the hoop stress and critical stress intensity of the given material are crucial. The diffusion-induced stress is governed by physical and material properties like particle diameter, state of charge, theoretical capacity, specific molar volume, yield strength, Young’s Modulus, etc. Since the lithiation process is diffusive, it is essential to compare different candidate electrode materials based on their material properties as recorded in Table 1.

In the present work, the parametric analysis was performed based on the selection of a quantity (parameter) that needed to be optimized. A set of constraints and a free variable were used to substitute the constraint in the parameter for optimization [25]. Five material indices (M) were created to express mechanical performance and fracture resistance.

#### Constraints and Free Variables:

In the mechanical parametric analysis of battery systems, it is important to set certain constraints to limit the degree of variability of the material indices for comparison of materials. Setting the average non-dimensional concentration profile to a constant allows all electrode particles to have the same amount of lithium ions diffused within them.

(29) $$\hat{c}\left(\hat{r}\right)=\frac{c}{c_{max}}=Cons$$

Setting time of diffusion or the particle radius as constant became slightly challenging as keeping equal radius particle seems to be a more apt decision from a manufacturing perspective. However, the time required for charging was crucial compared to the radius of the particle. Comparison based on equality of temporal coordinates was found more suitable in this situation.

(30) $$\hat{t}=\frac{tD}{r_{o}{}^{2}}$$

Considering the total time required for diffusion was kept constant, the following could be derived.

(31) $$r_{o}\propto \sqrt{D}$$

The radius emerges out to be the free variable which substitutes the diffusion coefficient in the material indices. Another constraint was obtained from the yield criterion.

(32) $$\sigma_{r}^{el}-\sigma_{\theta }^{el}=\frac{\Omega E}{3\left(1-\nu \right)}\left[\tilde{c}-\frac{3}{r_{p}{}^{3}}\int_{0}^{r_{p}}\tilde{c}r^{2}dr\right]\leq S_{y}$$

Since the integral term in Equation (32) became constant while substituting $\tilde{c}$ from Equation (29), the maximum concentration allowed became proportional to the yield stress.

(33) $$c_{max}\propto \frac{S_{y}\left(1-\nu \right)}{\Omega E}$$

For the fracture analysis, it was considered that the upper limit for the stress intensity factor was bound by the fracture toughness of the material.

(34) $$K_{I}\leq K_{1C}$$

## 3. Results and Discussion

During lithiation, the lithium-ion concentration is higher near the surface of the particle and decreases near the core which occurs due to the effect of Fick’s law of mass diffusion. The intercalation of lithium-ion with the electrode material causes it to expand proportionally to the relative concentration of lithium-ion. The surface of the particle tries to expand more during lithiation than the core. Expansion causes the surface to be under compression while the core remains under tension. During delithiation, the surface lithium-ion concentration is lower than the core. Hence, the surface contracts faster causing tension on the surface and compression in the core. In real world application, the deformation of the electrode material could be detected during testing and control stage of manufacturing or in development of newer electrode materials. The strain response of the electrode would vary under different rates of charging. The stress thus induced in the electrode to oppose this deformation would cause cracks to propagate and the electrode to fail. Electrode materials with strain exceeding the elastic limit would deform plastically. Under such loading conditions, the material would not relax back to its original shape upon unloading. This could be severely problematic for electrodes with higher elastic modulus, as cyclic shape deformation and high residual stress would lead to faster fatigue failure. On the other hand, elastic loading is commonly observed in electrode particles upon lithiation, where the strain is below the critical limit. An elastic-perfectly plastic chemical diffusion model was developed to perform the stress and fracture analysis during lithiation of different electrode materials. The electrode materials were compared based on their mechanical stability, ability to handle faster charging without yielding and higher fracture characteristics. The lithiation process was considered without effects of reaction or phase transformation. The merit indices were developed considering constant state of charge, charging time and temperature of 298 K. The equations for the stress analysis and merit indices were solved using MATLAB platform.

**Figure 2:**
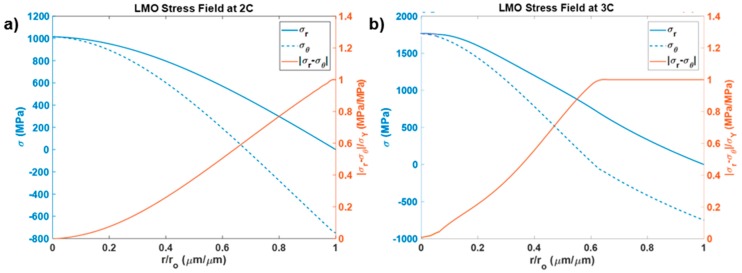
Stress distribution profile of a spherical lithium manganese oxide particle during lithiation under rates of charging of (**a**) 2C, and (**b**) 3C.

The mathematical analysis was done considering the electrode material to be elastic and perfectly plastic. Therefore, when the yield criteria (Equation (13)) was reached, the equivalent stress ($\left|\sigma_{r}-\sigma_{\theta }\right|$) remained equal to the yield stress of the material, while the material kept expanding plastically. The plastic deformability was experimentally observed by Kosova et al [41] and Schilcher et al [42]. In Figure 2, the normalized stress distribution was plotted against the normalized radius of a lithium manganese oxide particle. The particle was considered of 10µm in radius and charged under 2C and 3C rates of charging. For the 2C charging rate, the lithium manganese oxide particle was found barely plastic near the particle surface, where the equivalent stress equaled the yield stress of the material. However, with an increase in the charging rate to 3C, the electrode particle deformed plastically from the surface till about $0.65r_{0}$. The stress distribution in 2C case was found to be much more uniform, while for higher rate of charging, the stress profile became sharp in the plastic shell. This pushed the tensile stress domain in the core to a very high stress state. It is interesting to note that the stress (radial and hoop) developed 1.3 times and 2.4 times in magnitude of the yield stress for the 2C and 3C cases, respectively. However, the material did not fail under such high loads because the equivalent stress near the core was nearly zero. Hence, the material was under a purely tensile hydrostatic load which prevented failure because the electrode particle was considered to be solid without any crack. It could be inferred that the presence of small voids or microcracks in this domain, as observed experimentally in silicon [37], would lead to the voids to coalesce and it would form cracks. These cracks would propagate rapidly towards the surface and would get closed in the compressive domain near the surface. This would lead to failure of the electrode material above a certain dimension and rate of charging. It could then be inferred that lithium manganese oxide particles of 10µm radius are safe for operation under 2C charging rate.

### 3.1. Charging Index

Faster rate of charging is one of the most desired outcomes of battery research. Under the constraint of yield (Equation (33)), the boundary condition in Equation (8) was modified by substituting the flux from Equation (5) and elastic stress from Equation (25).

(35) $$-D\frac{\partial c}{\partial r}\left(1+\theta c\right)=\frac{i}{F}$$

Equation (35) was normalized with $\hat{c}=\frac{\tilde{c}}{c_{\max}}$, $\hat{r}=\frac{r}{r_{o}}$ and the current flux term was expanded from Equation (7).

(36) $$-\frac{\partial \hat{c}}{\partial r}\left(1+\theta c_{max}\hat{c}\right)=\frac{\rho \alpha r_{o}^{2}}{DFc_{max}}C_{rate}$$

Ignoring one in the left bracket and considering constraint of constant concentration and gradient from Equation (29), time constraint (Equation (30)) and approximation for maximum concentration from Equation (33), the rate of charging could be evaluated. The following merit index was minimized for materials that handles higher rates of charging.

(37) $$M_{Cr}=\frac{\rho E\alpha }{S_{y}^{2}{\left(1-\nu \right)}^{2}}$$

The charging rate merit index parameterized materials based on their ability to handle high charging rates without yielding. This index was developed with the constraint on the maximum lithium ion concentration that the electrode material could hold. Moreover, the time of complete lithiation remained constant for all the materials. A higher charging index indicated that the material that can store more charge in a lesser time without yielding, shows better promise as a future battery material for HEV battery systems. 

### 3.2. Elastic and Plastic Indices

The differential equations (Equations (26) and (27)) were related to stress-based diffusion through θ and Π which influenced the diffusion process. The elastic equation (Equation (26)) was promoted by θ (additive), while the plastic equation (Equation (27)) reduced with the increase of Π (subtractive). For lower stress response, it could be concluded that the concentration gradient during diffusion should be minimized for minimization of the following elastic merit index and the plastic merit index.

(38) $$M_{El}=\frac{\Omega^{2}Ec_{max}}{\left(1-\nu \right)}$$

(39) $$M_{Pl}=\frac{1}{S_{y}\Omega }$$

The diffusion equation (Equation (25)) got modified by the addition of extra concentration terms from the mean elastic stress field. These terms deviated the concentration distribution from its general parabolic structure to a higher gradient distribution, resulting in hindered diffusion in the particle. Higher gradient, or slower diffusion, therefore, leads to large stress build up and battery not being utilized to its utmost potential. It is crucial to understand the importance of this effect and to find ways to minimize its impact on the diffusion process.

### 3.3. Stress Index

Under elastic loading, the hydrostatic stress in Equation (25) was influenced by the concentration gradient in the particle. For small plastic stress effects, the equation was simplified.

(40) $${\hat{\sigma }}_{h}^{el}\propto \frac{\Omega Ec_{max}}{S_{y}\left(1-\nu \right)}\left[\frac{3}{{\hat{r}}_{p}{}^{3}}\int_{0}^{{\hat{r}}_{p}}\hat{c}{\hat{r}}^{2}dr-\hat{\tilde{c}}\right]$$

The stress was normalized by the yield stress and the integral part of the equation was considered constant (Equation 29 in 40). The following merit index for stress was minimized for prediction of low stress-induced materials.

(41) $$M_{St}=\frac{\Omega Ec_{max}}{S_{y}\left(1-\nu \right)}$$

### 3.4. Fracture Index

The fracture formulation (Equation (28)), with the hoop stress from Equation (25) (simplified for negligible yield stress) and the fracture toughness constraint (Equation (34)) was used to express the detectable crack length, which needed to be maximized for longer life. Therefore, the merit index was minimized in order to select a material with high fracture resistance.

(42) $$M_{Fr}=\frac{\Omega Ec_{max}}{K_{1C}\left(1-\nu \right)}$$

Material under stress tends to fail in the presence of cracks. Cracks or flaws act as stress concentrators which magnify the local stress field. If the crack size is beyond the critical limit, the stress at the crack tip exceeds the fracture strength of the material causing failure due to crack propagation.

**Figure 3:**
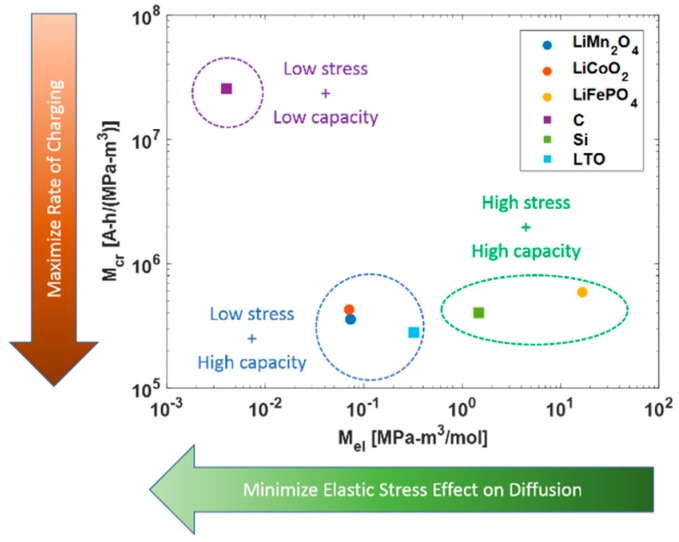
Material Selection based on Charging Rate Index and Elastic Stress Index.

Figure 3 demonstrated a multivariable comparison between the merit index based on maximizing the charging rate and the merit index for reducing the effect of elastic stress on diffusion. The objective of this comparison was selection of high capacity electrode materials to maintain lithium-ion storage capacity under high elastic deformation. The elastic merit index was dependent on the partial molar volume, modulus of elasticity and maximum concentration of lithium that the material can store. All these parameters were minimized for lower elastic effects on diffusion. 

The selected electrode materials, three for the cathode and three for the anode, were compared based on the formulated indices. Figure 3 shows that lithium manganese oxide was the most suitable material for the cathode and lithium titanate showed good promise for anode based on their high charge storage capability and low effects of elastic stress on lithium diffusion. Lithium manganese oxide was found experimentally more stable than commercially used lithium cobalt oxide [43]. The three-dimensional structure of the manganese oxide spinel allowed more space for intercalation with lithium ions during discharge and vice-versa for charge [9]. This means that manganese oxide allows faster lithiation without significant deformation. Silicon showed a tremendous performance compared to graphite, based on the ability to be operated at high charging rates [44]. However, graphite being soft generated lower elastic stresses during lithiation. While silicon did not perform well because of its high molar volume. Silicon expanded by 4 times its volume on lithiation leading to a severe effect on lithium diffusion during lithiation. Lithium titanate showed good performance amongst the anode material due to its higher yield strength and high capacity. The compared based on the ability to store charge silicon exceeded lithium titanate but its lower yield strength made it more prone to yielding than the later.

The charging index depends directly on the square of the yield strength of the material and inversely to the charge capacity and elastic modulus. Materials like lithium ferrous phosphate have a lower rating on this index. It is advisable to apply strengthening mechanism to improve the yield strength. Furthermore, lithium ferrous phosphate showed very poor characteristics on the elastic merit index scale because of its very high molar volume. This meant that ferrous phosphate electrodes deformed elastically more than lithium manganese oxide and lithium cobalt oxide electrodes. Newer materials could be hardened and/or alloyed to improve their performance. This would allow materials to be charged quicker without failure. Furthermore, the stress developed in the particle came from the mass flux of lithium ions. It was noted from Equation (7) that if the radius of the particle was reduced, it would allow higher charging rates for same flux. However, this is a tradeoff between manufacturability of smaller particles against the mechanical performance of the electrode.

**Figure 4:**
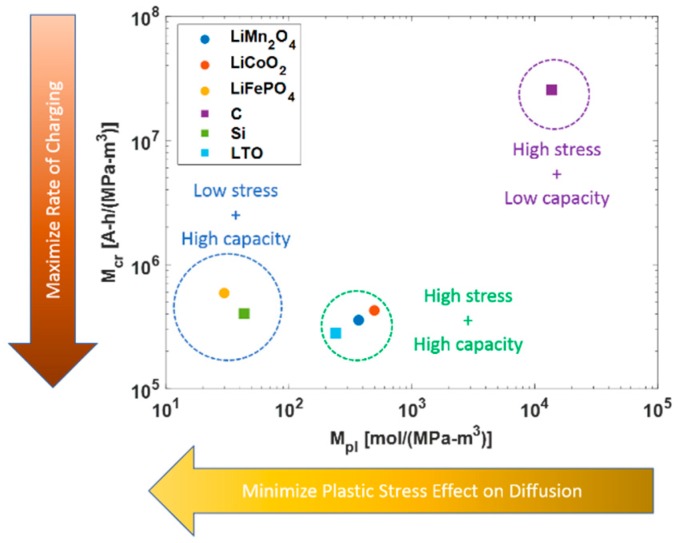
Material Selection based on Charging Rate Index and Plastic Stress Index.

As observed, faster charging increased the slope of the concentration profile in the electrode particle. It caused the equivalent stress of the particle to exceed the yield limits and the particle deforms plastically. Plastic mean stress also affected the diffusion process. However, the yield stress and molar volume reduced the concentration gradient and allowed free expansion of the particle. Therefore, it is required to maximize this scale for minimization of plastic deformation. Figure 4 compared the materials by maximizing the merit index for charging while minimizing the plastic deformation effect on diffusion. Lithium ferrous phosphate electrode was an excellent cathode material under this category in comparison to lithium manganese oxide and cobalt oxide. The merit index showed good the performance of silicon for the anode. Silicon’s ability to be lithiated under plastic deformation was observed experimentally [21], which validated the integrity of the plastic merit index. Graphite showed very poor plastic performance because of its low yield strength and brittle nature, making it vulnerable in the plastic deformation domain. Therefore, silicon is an excellent choice for HEV batteries which has very high capacities with the ability to operate under plastic deformation.

**Figure 5:**
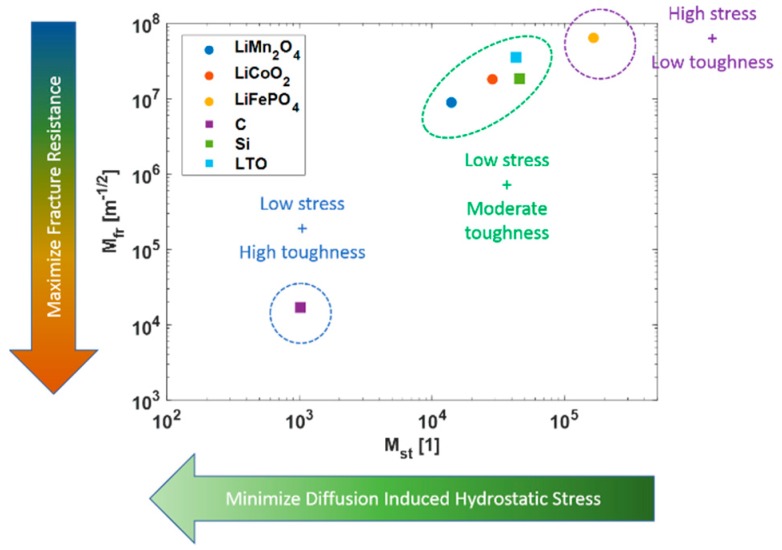
Material Selection based on Fracture Index and Hydrostatic Stress Index.

The strength of the material was compared against the material toughness. Figure 5 shows a comparison between the diffusion induced hydrostatic stress index against the fracture resistance index for different electrode materials. It could be inferred that higher yield strength means better mechanical performance, lower elastic modulus means a softer material which generates less stress during deformation, while lower molar volume means smaller volumetric deformation during lithiation. The study also showed that lithium manganese oxide showed the best mechanical performance as the cathode. The stress developed due to the concentration gradient, which is the prime source of stress, was minimum for lithium manganese oxide and was the highest for lithium ferrous phosphate. This was attributed due to the lower modulus of elasticity and molar volume of manganese oxide, which developed low stress and high fracture toughness, making it more resilient to fracture. Silicon was found comparably poor to graphite because of its low elastic modulus. However, silicon was comparable to other cathodic material making it the second-best candidate. Silicon can store more charge than graphite making this tradeoff favorable towards the former. To improve mechanical characteristics of new materials, it is important to reduce the molar volume and/or increase the yield strength. 

Different chemo-mechanical processes can harden the material making it durable. Doping the electrode material with a chemical reagent is an excellent way to alter the molar volume of the material. Alteration of the stoichiometry of the material reduces the distortion strains resulting in less expansion and improvement in fracture characteristics like toughness etc. Silicon, for example, can be made tougher by coating it with a more resilient inert layer like titanium oxide [45]. The selection of material under high charging rates depends on their mechanical performance and fracture stability. Higher mechanical stability of material having higher yield strength can be achieved by toughening the material. Size of the electrode particle also plays a crucial role in determining its performance. Smaller particles can be charged faster for the same stress and they allow better diffusion. It also leads to the lower concentration gradient of lithium-ion and consequently better mechanical stress characteristics. Silicon has a very high charge capacity which makes it a good choice for high charging systems where plastic deformation is dominant. However, its fracture characteristics need to be improved for a safer design.

## 4. Conclusions

The paper discussed an elasto-plastic based diffusion-induced stress model and a set of five material indices for material categorization and selection. The model was used to determine the concentration profile for lithium-ion in the elastic and plastic domain. The concentration was used to find the stress profile for the electrode during lithiation. The comparison between the stress profile for lithium manganese oxide under 2C and 3C charging rates showed that the center of the particle was under high tensile hydrostatic loading during lithiation. The equivalent stress being zero at the core prevents failure of the particle. However, the presence of voids would lead to failure by crack nucleation and propagation over multiple cycles. The materials were evaluated based on their charge holding capability under elastic and plastic loading. It was found that under elastic loading conditions, lithium manganese oxide and graphite are the best cathode and anode materials while under plastic loading, lithium ferrous phosphate and silicon are the best material choice for the battery. The strength of the electrode material was compared against its toughness, and lithium manganese oxide and graphite showed the best performance. From the material parameterization, it was inferred that lithium manganese oxide was the most suited cathode material due to its ability to perform well under faster charging and showing excellent mechanical and fracture characteristics. Graphite performed great in handling elastic stress and was resistant to fracture. However, newer materials like silicon and lithium titanate were found to be good for faster charging and in handling plastic deformation. This made them good choices for HEV battery modules. Furthermore, newer materials could be made better by lowering the elastic modulus and molar volume and improving the yield strength by toughening. Fracture toughness could be improved by coating the material with a more resilient substance to absorb the fracture energy. Reducing particle size would be a great alternative, as it would allow higher rates of charging for same current flux and lower mechanical stresses. These indices have a great importance in classifying good electrode materials and aid in the search for newer battery materials.

## Figures and Tables

**Table 1:** Material properties of selected electrode materials.

Properties	LiMn_2_O_4_	LiCoO_2_	LiFePO_4_	Li_x_C_6_	Li_x_Si_15_	Li_x_TiO_2_
$D\;\left(m^{2}/s\right)$	7.08 × 10^−15^ [17]	1.00 × 10^−13^ [26]	7.96 × 10^−16^ [27]	3.90 × 10^−14^ [26]	1.00 × 10^−16^ [28]	6.80 × 10^−15^ [29]
ρ (kg/m)	4100 [17]	5030 [26]	3600 [30]	2100 [26]	2328 [31]	3510 [32]
$\alpha_{th}\;\left(mAh/g\right)$	148 [33]	166 [33]	170 [33]	372 [33]	4200 [33]	175 [7]
$c_{max}\;\left(mol/m^{3}\right)$	2.29 × 10^4^ [17]	4.99 × 10^4^ [26]	2.12 × 10^4^	3.05 × 10^4^ [26]	8.87 × 10^4^ [34]	5.00 × 10^4^ [29]
$\Omega \;\left(m^{3}/mol\right)$	3.50 × 10^−^^6^ [17]	1.92 × 10^−^^6^ [26]	67.32 × 10^−^^6^ [35]	3.17 × 10^−^^6^ [26]	32.25 × 10^−^^6^ [36]	5.00 × 10^−^^6^ [29]
$S_{y}\;\left(MPa\right)$	776 [37]	1056 [38]	500 [38]	23 [33]	720 [21]	836
E (GPa)	194 [38]	264 [38]	125 [38]	10 [38]	12 [39]	209 [38]
Ν	0.26 [38]	0.32 [38]	0.28 [38]	0.24 [38]	0.25 [38]	0.19 [38]
$K_{1C}\;\left(MPam^{0.5}\right)$	1.50 [40]	1.30 [40]	1.50 [40]	1.25 [40]	1.00 [40]	1.50 [40]
